# Invited Response on: ‘Comment on: A Novel Method of Outcome Assessment in Breast Reconstruction Surgery: Comparison of Autologous and Alloplastic Techniques Using Three-Dimensional Surface Imaging’

**DOI:** 10.1007/s00266-020-02026-0

**Published:** 2020-11-02

**Authors:** Vanessa Brébant, Robin Hartmann, Lukas Prantl

**Affiliations:** University Center of Plastic, Aesthetic, Hand and Reconstructive Surgery, University Hospital Regensburg, Franz-Josef-Strauß-Allee 11, 93053 Regensburg, Germany

*Level of Evidence V* This journal requires that authors assign a level of evidence to each article. For a full description of these Evidence-Based Medicine ratings, please refer to the Table of Contents or the online Instructions to Authors www.springer.com/00266.

We thank the authors for their interest in our article “A Novel Method of Outcome Assessment in Breast Reconstruction Surgery: Comparison of Autologous and Alloplastic Techniques Using Three-Dimensional Surface Imaging” [1].

During the last 2 years, we have worked extensively on improving the outcome assessment in breast reconstruction using three-dimensional surface imaging. The result is an independent software, which uses standard 3D file formats to assess breast symmetry by digital anthropometry.

In this trial, we used this novel software, designed for patients who underwent breast reconstruction surgery (BRS), by comparing two surgical breast reconstruction techniques.

We agree that the methodology of comparing successful alloplastic and autologous techniques is appealing, and we thank the authors for their appreciation.

In the article, we mention that we investigated all patients who underwent reconstruction from January 2015 to January 2018, a period of 3 years.

We have to clarify that the complication rate the authors mention is incorrect. As stated in our manuscript, 118 patients out of an initial cohort of 183 patients undergoing BRS at the study center during the observed study period were randomly selected. They were selected independent of any surgical complications. We selected alloplastic and autologous reconstructions by equal amounts. As the purpose of the present trial was to compare outcomes in successful BRS, all patients with flap loss or implant loss were then excluded.

We investigated the complication rate at our institution in a separate study. The results, published in 2020, showed one flap loss in 44 autologous reconstructions [2]. In another study, we compared two distinct mastectomy techniques followed by autologous reconstruction with regard to breast sensitivity [3].

The present trial’s objective was to compare the outcomes in successful BRS and not to investigate the complication rates of different procedures in BRS.

We concluded that in our trial no differences in the outcomes’ optical symmetry were found. The conclusion, we believe, is justified. We agree that the study’s sample size was small. However, we have identified and extensively discussed this study’s limitations.

In previous investigations, we identified features that determine female bodily attractiveness [4, 5]. We appreciate the authors’ references to their review on the metrics of the ideal breast [6].

We are currently conducting a prospective study using our prototype software for patients who underwent BRS, including numerous subgroups. We have already incorporated improvements based on the present study’s findings. These are in concordance with the authors’ advice.

We considered the authors’ suggestion to test our prototype software on non-operated healthy women and thank them for their recommendations for improvement.

With our data, we aspire to provide a foundation to use 3D imaging of the breast region with regard to BRS to support both the patients and the board-certified plastic surgeons. Today, the field of plastic surgery is leading at implementing this avant-garde technology. With our prototype software, we are trying to contribute to the advancement of outcome assessment in BRS.

## References

[1] Hartmann R, Weiherer M, Schiltz D, Seitz S, Lotter L, Anker A (2020). A novel method of outcome assessment in breast reconstruction surgery: comparison of autologous and alloplastic techniques using three-dimensional surface imaging. Aesthet Plast Surg.

[2] Anker AM, Prantl L, Strauss C, Brébant V, Schenkhoff F, Pawlik M (2020). Assessment of DIEP flap perfusion with intraoperative indocyanine green fluorescence imaging in vasopressor-dominated hemodynamic support versus liberal fluid administration: a randomized controlled trial with breast cancer patients. Ann Surg Oncol.

[3] Heine N, Koch C, Brebant V, Kehrer A, Anker A, Prantl L (2017). Breast sensitivity after mastectomy and autologous reconstruction. Clin Hemorheol Microcirc.

[4] Gründl M, Eisenmann-Klein M, Prantl L (2009). Quantifying female bodily attractiveness by a statistical analysis of body measurements. Plast Reconstr Surg.

[5] Prantl L, Gründl M (2011). Males prefer a larger bust size in women than females themselves: an experimental study on female bodily attractiveness with varying weight, bust size, waist width, hip width, and leg length independently. Aesthet Plast Surg.

[6] Atiye B, Chahine F (2018). Metrics of the aesthetically perfect breast. Aesthet Plast Surg.

